# EM011 activates a survivin-dependent apoptotic program in human non-small cell lung cancer cells

**DOI:** 10.1186/1476-4598-8-93

**Published:** 2009-10-30

**Authors:** Prasanthi Karna, Starlette M Sharp, Clayton Yates, Satya Prakash, Ritu Aneja

**Affiliations:** 1 Department of Biology, Georgia State University, Atlanta, GA-30303, USA; 2 Department of Biology and Center for Cancer Research, Tuskegee University, Tuskegee, AL-36088, USA; 3 Department of Biomedical Engineering, McGill University, Montreal, H3A 2B4, Canada

## Abstract

**Background:**

Lung cancer remains a leading cause of cancer death among both men and women in the United States. Treatment modalities available for this malignancy are inadequate and thus new drugs with improved pharmacological profiles and superior therapeutic indices are being continually explored. Noscapinoids constitute an emerging class of anticancer agents that bind tubulin but do not significantly alter the monomer/polymer ratio of tubulin. EM011, a rationally-designed member of this class of non-toxic agents, is more potent than the lead molecule, noscapine.

**Results:**

Here we report that EM011 inhibited proliferation of a comprehensive panel of lung cancer cells with IC_50_'s ranging from 4-50 μM. In A549 human non-small cell lung cancer cells, the antiproliferative activity was mediated through blockage of cell-cycle progression by induction of a transient but robust mitotic arrest accompanied by activation of the spindle assembly checkpoint. The mitotically-arrested A549 cells then override the activated mitotic checkpoint and aberrantly exit mitosis without cytokinesis resulting in pseudo G1-like multinucleated cells that either succumb directly to apoptosis or continue another round of the cell-cycle. The accumulated enormous DNA perhaps acts as genotoxic stress to trigger cell death. EM011-induced apoptotic cell death in A549 cells was associated with a decrease of the Bcl2/BAX ratio, activation of caspase-3 and cleavage of PARP. Furthermore, EM011 induced downregulation of survivin expression over time of treatment. Abrogation of survivin led to an increase of cell death whereas, overexpression caused decreased apoptosis.

**Conclusion:**

These *in vitro *data suggest that EM011 mediates antiproliferative and proapoptotic activity in non-small cell A549 lung cancer cells by impeding cell-cycle progression and attenuating antiapoptotic signaling circuitries (viz. Bcl2, survivin). The study provides evidence for the potential usefulness of EM011 in chemotherapy of lung cancer.

## Background

Lung cancer is a leading cause of death worldwide. Non-small cell lung cancer (NSCLC) accounts for ~80-85% of all cases of lung cancer, and ~45% of patients present with stage IIIA/B disease [1]. Besides the metastatic nature of this disease, drug resistance that emerges upon prolonged treatment with particular drug/s has been responsible for poor survival statistics, and the overall scenario emphasizes need for effective and well-tolerated treatment regimens. Even with the best currently-available treatment, lung cancer can only be cured at its earliest stage, and the 5-year survival rate is a low 5 percent. Although many traditional cytotoxics have been used as monotherapy in NSCLC, including vindesine, docetaxel, carboplatin, etoposide, ifosfamide, cyclophosphamide, vincristine, mitomycin and cisplatin [2], these drugs produce only small improvements, and several debilitating toxicities significantly compromise the quality of life and decrease survival. Thus, the need for development of more effective therapeutic strategies for NSCLC that offer improved pharmacological profiles and superior therapeutic indices is crucial.

The mitotic spindle, a highly evolved elegant structure that orchestrates faithful chromosome segregation during cell division, is a pharmaceutically validated target for anticancer therapy [3,4]. Since dynamic microtubules that compose the mitotic spindle have a critical role in cell division, various microtubule inhibitors have been developed as successful anticancer drugs. Two major classes of microtubule-interfering agents are well recognized in the clinic today. They comprise the *taxanes *(represented by paclitaxel, docetaxel etc.) that overpolymerize and bundle microtubules, and the *vinca alkaloids *(typified by vinblastine, vincristine, vinflunine etc.) that depolymerize microtubules. Several of these microtubule depolymerizing agents have been widely employed for the treatment of NSCLC [5,6]. However, due to the extreme effects of these drugs on microtubules, critical physiological functions that microtubules perform, such as intracellular transport, are compromised (reviewed in [7]). In addition, these microtubule inhibitors act on both proliferating and post-mitotic cells and thus exhibit microtubule-dependent side effects, including peripheral neuropathy [8,9].

Noscapinoids, an emerging class of microtubule-modulating anticancer agents based upon the lead molecule, noscapine apparently avoid the harsher effects of the currently-available antimicrotubule agents [10-19]. Noscapine and its analogs do not alter the steady state polymer levels of tubulin, instead dampen microtubule dynamics just enough to perhaps activate the mitotic checkpoints to halt mitosis without perturbing other vital microtubule functions such as axonal transport [13,16]. This perhaps might be the reason for lack of apparent toxicity upon treatment with noscapine and its analogs [11,14-18]. Based upon anticancer activity and non-toxic attributes, the parent molecule, noscapine, is already in Phase I/II clinical trials.

The brominated noscapine analog, EM011, is more active than the parent noscapine, as reported by the 60-cell line anticancer screen conducted by the Developmental Therapeutics Program (DTP) at the National Institutes of Health [19]. EM011 retains the tubulin-binding capacity and preserves the non-toxic attributes of noscapine [13-15,17,18]. Earlier reports have shown that EM011 inhibited growth of pgp- and MRP- overexpressing human lymphoma xenografts implanted in nude mice [14,17]. In this study, we wished to evaluate whether or not EM011 that shows potent *in vitro *and *in vivo *anticancer activity in several cancer types, is useful for treating lung cancer. Our results demonstrate that EM011 inhibited proliferation of a variety of human lung cancer cells with an IC_50 _ranging from 4-50 μM. A robust mitotic arrest through activation of the spindle assembly checkpoint was observed upon drug-treatment of A549 cells. Mitotically-arrested cells then exited mitosis without cytokinesis in a phenomenon called mitotic slippage into a pseudo G1-like interphase state and subsequently activated apoptotic cell-death pathways. However, a small percentage of these mitotically-slipped tetraploid cells entered another round of cell-cycle and thus accumulated massive DNA amounts that possibly trigger apoptosis due to genotoxic stress. Furthermore, some multinucleate cells that are perhaps 'apoptosis-reluctant' undergo aberrant cell divisions into several (more than 2) cells suggesting induction of aneuploidy. At the molecular level, EM011 downregulated survivin, an important member of the inhibitor of apoptosis (IAP) family of proteins. Abrogation of survivin by small-interfering RNA led to an increased sensitivity of A549 cells towards EM011-induced cell death. These results suggest the potential usefulness of EM011 in the management of lung cancer and warrant further evaluation.

## Materials and methods

### Chemicals and reagents

EM011 ((S)-3-((R)-9-bromo-4-methoxy-6-methyl-5,6,7,8-tetrahydro-[1,3]dioxolo [4,5-g]isoquinolin-5-yl)-6,7-dimethoxyisobenzofuran-1(3H)-one) was synthesized as described earlier [13,19]. Sulforhodamine B (SRB), 4-6-diamidino-2-phenylindole (DAPI), propidium iodide (PI), RNase, bovine serum albumin (BSA), and the mouse monoclonal antibody against α-tubulin were from Sigma-Aldrich (St. Louis, MO, US). Antibodies against caspase-3, cleaved PARP, Bcl2, BAX were obtained from Cell Signaling (Beverly, MA). BubR1 was purchased from BD Biosciences, PharMingen. Horseradish peroxidase-conjugated anti-rabbit and anti-mouse secondary antibodies were purchased from Sigma. Fluorescein-conjugated anti-mouse antibody was from Jackson ImmunoResearch, Inc. (West Grove, PA).

### Cell lines and plasmids

Human lung cancer cells (H322 M, H266, H266B, H23, H460, HOP-92, HOP-62, H1299, A549, H522, H157, and H1792) were obtained from Dr. Wei Zhou's laboratory, Winship Cancer Institute, Emory University. All cells were maintained in RPMI-1640 media supplemented with 10% fetal bovine serum (Invitrogen Carlsbad, CA) and 1% antibiotics. The plasmid construct encoding survivin and survivin siRNA plasmid were from Dr. Lily Yang, Winship Cancer Institute, Emory University. The plasmid vector with survivin siRNA sequence 5'-GGCTGGCTTCATCCACTGCCC-3' was generated by cloning the synthesized oligonucleotide into pSilencer 2.1-U6Neo plasmid (Ambion Inc., Austin, TX). Control pSilencer 2.1-U6 Neo plasmid vector containing a scrambled siRNA sequence, 5'-ACTACCGTTGTTATAGGTGT-3', was obtained from Ambion Inc. The survivin plasmid contruct was cloned into the pcDNA3 vector and pcDNA3 vector alone served as control. All transfections were done using Lipofectamine 2000 following the manufacturers' instructions.

### In vitro cell proliferation assay

Lung cancer cells were seeded in 96-well plates at a density of 5 × 10^3 ^cells/well followed by next day treatment with increasing gradient concentrations of EM011 ranging from 10 nm to 100 mM. After 48 hrs of drug treatment, cells were fixed with 50% trichloroacetic acid and stained with 0.4% sulforhodamine B (SRB) dissolved in 1% acetic acid. Cells were then washed with 1% acetic acid to remove the unbound dye. Essentially, the SRB assay measures cell density by quantitating colored SRB bound to cellular proteins fixed to the plates by tricholoroacetate. The protein-bound dye was extracted with 10 mM Tris-base to determine the absorbance at 564 nm wavelength [20,21]. The percentage of cell survival as a function of drug concentration was then plotted to determine IC_50 _values (drug concentration needed to prevent cell proliferation by 50%).

### Cell-cycle analysis

A549 cells were seeded in culture dishes and grown until ~70% confluence. The medium was then replaced with new medium containing either vehicle (0.01% DMSO) or 25 μM EM011 for 6, 12, 18, 24, 36 and 48 hrs. After the incubation period, cells were centrifuged, washed twice with ice-cold PBS, and fixed in 70% ethanol. Tubes containing the cell pellets were stored at 4°C for at least 24 hrs. Cells were then centrifuged at 100 × g for 10 minutes, and the supernatant was discarded. Pellets were washed twice with PBS and stained with PI in the presence of RNase A for 45 minutes in dark. Samples were analyzed on a FACSCalibur flow-cytometer (Beckman Coulter, Inc., Fullerton, CA).

### Quantitation of mitotic cells by MPM-2 staining

A549 cells were collected and fixed with 70% ethanol. Cells were treated with the blocking solution (PBS containing 1% Triton X-100 and 1% BSA) for 1 hr, followed by incubation with MPM-2 mouse monoclonal antibody (Upstate Biotechnology, Lake Placid, NY) in blocking solution for 1 hr. Cells were then incubated with Alexa 488-conjugated anti-mouse secondary antibody followed by PI for DNA staining, and were read flow-cytometrically.

### Immunofluorescence confocal microscopy

A549 cells were grown on poly(L-lysine)-coated glass coverslips for immunofluorescence microscopy as described previously [13,15]. After treatment, cells were fixed with cold (-20°C) methanol for 5 minutes and blocked by incubating with 2% BSA/PBS at 37°C for 1 hr. A mouse monoclonal antibody against α-tubulin (DM1A, Sigma) was diluted 1:500 in 2% BSA/PBS and incubated with the coverslips for 2 hrs at 37°C. Cells were then washed with 2% BSA/PBS for 10 minutes at room temperature before incubating with a 1:200 dilution of a FITC-labeled goat anti-mouse IgG antibody (Jackson ImmunoResearch, Inc., West Grove, PA) at 37°C for 1 hr. Coverslips were then rinsed with 2% BSA/PBS for 10 minutes and incubated with PI (0.5 μg/mL) for 15 minutes at room temperature before they were mounted with Aquamount (Lerner Laboratories, Pittsburgh, PA) containing 0.01% 1,4-diazobicyclo(2,2,2)octane (Sigma). Cells were examined using confocal microscopy for microtubule and nuclear morphology using a 63× objective (numerical aperture, 1.4).

### Western blot analysis

Proteins were resolved by polyacrylamide gel-electrophoresis and transferred onto polyvinylidene difluoride membranes (Millipore). Membranes were blocked in Tris-buffered saline containing 0.2% Tween-20 and 5% fat-free dry milk and incubated first with primary antibodies and then with horseradish peroxidase-conjugated secondary antibodies. Specific proteins were visualized with enhanced chemiluminescence detection reagent according to the manufacturer's instructions (Pierce Biotechnology).

### Terminal deoxynucleotidyl-transferase-mediated dUTP nick-end labeling (TUNEL) assay for apoptosis

DNA strand breaks were identified using the TUNEL assay as described [15]. Briefly, A549 cells treated with 25 μM EM011 for 72 hrs were washed with ice-cold PBS, fixed in 1% paraformaldehyde, and 3'-DNA ends were detected using the APO-BrdU TUNEL Assay Kit (Molecular Probes, Eugene, OR).

### Determination of caspase-3 activity

10^6 ^cells were incubated with 25 μM EM011 for 0, 12, 24, 48 and 72 hrs. Caspase-3 activity was measured by cleavage of the small synthetic substrate Z-DEVD-aminoluciferin (CaspaseGloTM 3/7 Assay System Kit, Promega, Madison, WI) that becomes luminogenic upon cleavage. The luminescent signal, which is directly proportional to the amount of caspase-3 activity, was measured in a luminescence plate reader.

## Results and Discussion

### EM011 inhibits growth of a wide array of lung cancer cells

**Figure 1:**
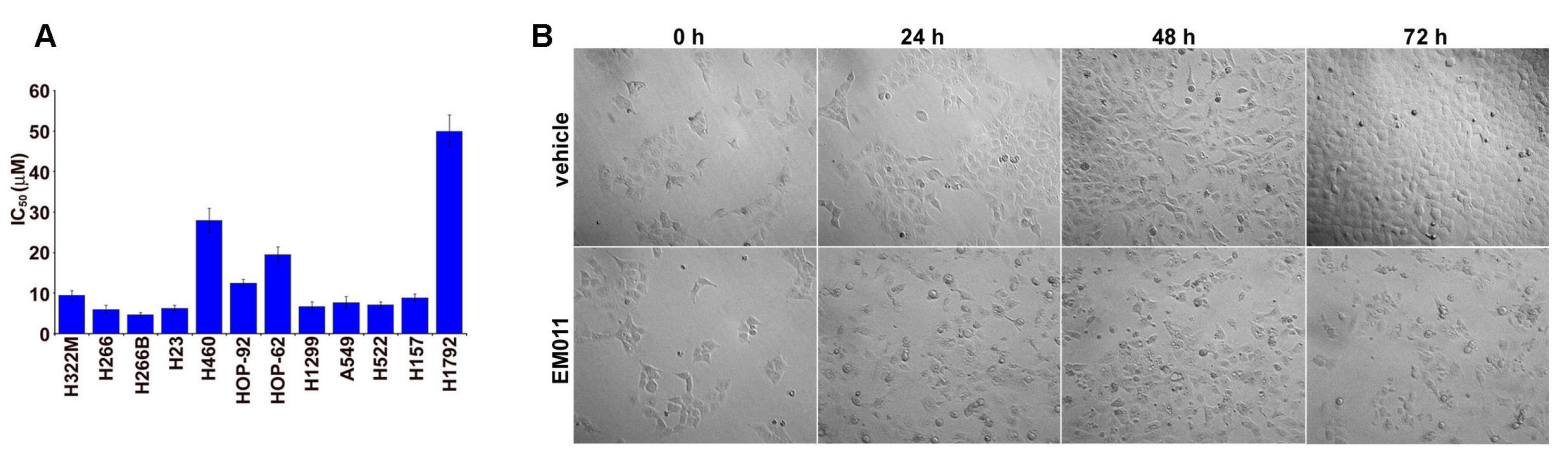
**EM011 suppresses proliferation of human lung cancer cells**. **A**. A panel of lung cancer cells ((H322 M, H266, H266B, H23, H460, HOP-92, HOP-62, H1299, A549, H522, H157, and H1792) were treated with gradient concentrations of EM011 for 72 hrs and the IC_50 _values were evaluated by the sulforhodamine B (SRB) *in vitro *cell proliferation assay. The values and error bars shown in the graph represent average and standard deviations, respectively, of three independent experiments (p < 0.05). **B**. Phase-contrast images (10×) of A549 cells treated with 25 μM EM011 for 0, 24, 48 and 72 hrs.

Noscapinoids represent a new generation of anticancer agents that modulate microtubule dynamics but do not significantly alter the total polymer mass of tubulin. Earlier reports have shown that EM011, a brominated noscapine analog, has potent antiproliferative and proapoptotic activity in hormone-refractory breast cancer and drug-resistant lymphoma models [14,15,17]. To evaluate the efficacy of EM011 in lung cancer cells, we first examined the ability of EM011 to inhibit cellular proliferation in a comprehensive panel of lung cancer cells with variable but well-characterized genotypes. The panel included H322 M, H266, H266B, H23, H460, HOP-92, HOP-62, H1299, A549, H522, H157, and H1792 lung cancer cells. These 12 different lung cancer cell lines were treated with gradient concentrations of EM011 and the extent of cell proliferation was measured by the SRB assay, which is based on the stoichiometric binding of SRB dye to all cellular protein components [21]. As shown in Figure 1A, EM011 effectively suppressed cellular proliferation of lung cancer cells. The non-small cell lung cancer cell lines included in the study had well-characterized p53 status, namely, wild type (represented by H226, H460, A549), null (such as H1299) and mutant (H322 M, H23, H522, H157, H1792). Although the parent molecule, noscapine, has been previously shown to induce a p53-dependent apoptosis in colon cancer cells [22], these data show that the half-maximal growth inhibitory concentrations of EM011 did not correlate with the p53 status in lung cancer cells. For example, H1299 that lacks endogenous p53 showed similar sensitivity as A549 (wild type p53) or H522 (mutant p53) cells. The IC_50 _values for most lung cancer lines studied were in the range of 4-10 μM. The relatively less-sensitive lung cancer cells included HOP-92 (IC_50 _= ~12.5 μM), HOP-62 (IC_50 _= ~19.5 μM), H460 (IC_50 _= ~28 μM) and H1792 (IC_50 _= ~50 μM). Since the A549 human epithelial cell line is widely-accepted as a standardized experimental model with biological properties of alveolar epithelial type II cells, we chose these cells to conduct further studies to gain insights into cellular and molecular mechanisms of EM011 action. Phase-contrast microscopic analysis of cell morphology showed that while vehicle-treated A549 cells proliferated normally, EM011 treatment impaired their proliferation capacity (Figure 1B). Cells first appeared rounded-up (24 hrs treatment) followed by a fragmented morphology (72 hrs treatment) (Figure 1B).

### EM011 transiently blocks cell-cycle progression in the G2/M phase

**Figure 2:**
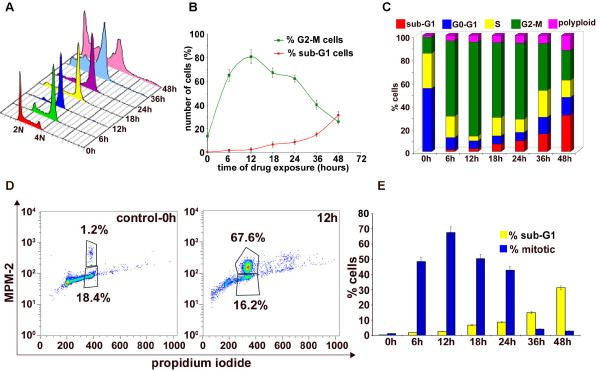
**EM011 perturbs cell-cycle progression of A549 lung cancer cells**. Cells were harvested for analysis at the noted times, fixed and stained with propidium iodide, and analyzed by flow-cytometry using the Cell Quest Software. **A**. Effect of 25 μM EM011 on cell-cycle progression of A549 cells over time shown in a three-dimensional disposition. X-axis, intensity of propidium iodide fluorescence, is indicative of the total DNA content of cells in various phases of the cell-cycle. Y-axis, number of cells detected for a given DNA content. Z-axis, time points (i.e., 0, 6, 12, 18, 24, 36, and 48 hrs). Representative results of three independent experiments. **B**. Quantitative graphical representation of the percentage of G2/M and sub-G1 cell population. Points, average of three independent experiments; bars, SD (p < 0.05). **C**. A bar-graphical representation of relative percentage of cells in various cell-cycle phases over time of drug treatment. A549 cells achieved a maximal G2/M population at 12 hrs (shown in green). **D**. Representative dot-plots depicting mitotic population at 0 and 12 hrs of drug-treatment as quantitated by MPM-2 and PI staining, flow-cytometrically. MPM-2, a well known phospho-serine/threonine antibody is recognized for its versatility because it can detect several mitotic phospho-proteins that are phosphorylated either directly or indirectly by M-phase-promoting factor (MPF) upon entry into mitosis. **E **is a bar-graphical representation highlighting the near inverse-correlation between the mitotic and sub-G1 population of cells in A549 cells. The values and error bars shown in the graph represent the averages and standard deviation (SD), respectively, of three independent experiments; (p < 0.05).

To investigate the precise mechanism responsible for EM011-mediated antiproliferative effects, we examined the cell-cycle distribution profile of EM011-treated A549 lung cancer cells over time. A flow-cytometric assay using the DNA intercalator dye, propidium iodide (PI), was utilized to monitor cell-cycle progression on the basis of status of DNA amounts. Figure 2A shows the time-dependent effects of EM011 on cell-cycle profile of A549 cells in a three-dimensional representation. The x-axis shows amount of DNA depicting different phases of the cell-cycle. While 2N and 4N DNA complements represent G0/G1 and G2/M cell populations respectively, S phase is characterized by variable DNA (between 2N and 4N) and sub-G1 population is usually indicative of degraded DNA, a hallmark of apoptosis. The y-axis represents the number of cells containing that amount of DNA and the z-axis shows the time of drug-exposure. 0 hr depicts cell-cycle profile of control cells. As we go along in time, EM011 treatment caused a significant inhibition of cell-cycle progression in A549 cells resulting in an accumulation of cells in the G2/M phase compared to control cells (Figure 2B). The G2/M population began to sharply rise as early as 6 hrs (~65%) post-treatment, achieved a maximum at 12 hrs (~80%) and was still about ~60% at 24 hrs of EM011 exposure (Figure 2B). This increased population of cells with 4N DNA perhaps correlated with concomitant losses from G0/G1 phases (Figure 2B). Following this, a disappearance of the G2/M population and an emergence of a characteristic hypodiploid DNA content peak (sub-G1) was observed beginning 24 hrs, indicative of apoptotic cells (Figure 2B). The sub-G1 population at 36 hrs (~15%) increased to ~32% at 48 hrs post-treatment. Figure 2C is a bar-graphical representation that provides an overview of the percent cell population in various cell-cycle phases upon EM011 treatment over time. These results suggest that EM011-treated A549 cells arrested in the G2/M phase preceding cell death.

### EM011 treatment selectively accumulates cells in mitosis and not G2 phase

Although the cell-cycle experiment using PI for DNA read-out is extremely useful for an overview of the percent cell population in various cell-cycle phases, it cannot dissect out differences between G2 and M phases as both have 4N DNA amounts. Thus, we next examined the specific cell-cycle phase (G2 or M) in which the cells arrested upon EM011 treatment using a mitosis-specific marker, MPM-2. The MPM-2 positive (mitotic) population increased to ~48% (47.4 ± 2.3%) in 6 hrs and peaked to ~68% (67.6 ± 1.9%) at 12 hrs after which a decrease in the MPM-2 population was observed (Figure 2D). The MPM-2 positive population was still as high as ~43% (43.2 ± 2.1%) at 24 hrs. However, the mitotic population declined sharply to ~4% (3.8 ± 0.5%) at 36 hrs (Figure 2E).

### EM011 perturbs spindle architecture and interferes with bipolar spindle formation

**Figure 3:**
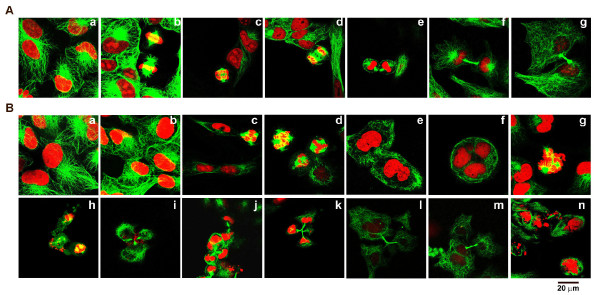
**A. Confocal immunomicrographs of microtubules (green) and DNA (red) in vehicle (0.01% DMSO) treated A549 cells displaying hallmarks of a typical cell division process in a cell-cycle, in particular, interphase (a), metaphase (b), early anaphase (c), late anaphase (d), telophase (e), and cytokinesis (f, g)**. **B**. In contrast, EM011 treatment arrested A549 cells at mitosis, impaired bipolar spindle formation, induced mitotic slippage, aneuploidy and apoptosis. EM011 treatment did not affect the radial array network of microtubules until 6 hrs of exposure (0 hr, (a); 6 hrs (b)). Aberrantly arrested cells with multipolar spindles were seen at 12 hrs (c) and 24 hrs (d) post-treatment. Cells started to exit mitosis without cell division beginning at about 30 hrs post-treatment (e). Thereafter, at 36-40 hrs post-treatment, several aberrant phenotypes were observed suggesting variability in cellular fate upon drug treatment. Multinucleate cells with small fragments of DNA indicating apoptotic bodies suggested cell death from G1-like tetraploid state (f). Some cells showed signs of DNA fragmentation while in mitosis suggesting mitotic death (g, h). However, some cells were seen to undergo asymmetric chaotic cytokinesis with triple midbody (i) and perhaps, led to the generation of aneuploid cells (j, k). Occasionally, aberrant separation into more than two abnormal cells (even multinucleated) were seen (l, m). At 48 hrs of drug exposure, several fragmented DNA pieces and clusters of small apoptotic bodies were observed (n).

We next visualized cellular microtubules and the spindle apparatus that is composed of microtubules in the absence and presence of EM011 to detect cellular events that were perturbed by drug action (Figure 3). The nuclear morphology of A549 cells was also examined over time of treatment. Immunofluorescence confocal microscopy was employed to examine drug effects-microtubules were stained green (FITC-labeled secondary antibody) and DNA was stained red using PI. Figure 3A represents confocal micrographs of A549 cells that were treated with vehicle (0.01% DMSO). Interphase cells showed normal radial arrays of microtubules (Figure 3A, a). The doubling time of A549 cells was observed to be about 22-24 hrs. The mitotic population, a small percentage (~6-10%) of the total number of cells in a cell-cycle at any given time, displayed hallmark features of a typical mitotic process. Congression of chromosomes at the metaphase plate followed by anaphase onset, a characteristic telophase and cytokinesis were evident (Fig 3A, b-g). Although EM011-treated cells at 0 and 6 hrs showed normal microtubule arrays (Figure 3B a, b), mitotically-arrested cells were visible at 12 hrs post-treatment (Figure 3B, c) and continued to accumulate until 24 hrs (Figure 3B, d). The arrested cells did not show normal bipolar spindles, rather displayed aberrant multipolar spindles. Predominantly, the number of spindle poles observed was 3 or 4 (Fig 3B, c, d). The mitotic block triggered by the spindle assembly checkpoint is not permanent, and cells can exit mitosis aberrantly by a process known as mitotic slippage (also known as adaptation) [23]. At 30 hrs post-treatment, there was an emergence of G1-like interphase cells that were multinucleated. Most likely, these multinucleated cells resulted from a mitotic exit, that is, cells slipped out of an abnormal multipolar mitosis without cell division (Figure 3B, e). Upon mitotic slippage, cells enter pseudo G1-like interphase with tetraploid (4N) cells. We observed that these multinucleate cells continued to accumulate until 36-40 hrs. At 40 hrs post-treatment, several phenotypes were observed, perhaps arising from the variability in cellular fates within the same cell line. This was in conformity with recent reports that suggest the existence of a high degree of inter- and intra- line variability in cellular responses to chemotherapeutic drug treatment [24,25]. Multinucleated cells with small pieces of fragmented DNA, reminiscent of apoptotic bodies, were also seen at about 40 hrs post-treatment suggesting initiation of cell death directly from the G1-like multinucleate state (Figure 3B, f). Perhaps, there was a small percentage of mitotic cells that were disintegrating, suggesting death directly from a prolonged mitotic arrest (Figure 3B, g, h). Interestingly, at 40-48 hrs of drug exposure, some multinucleate A549 cells (10-15%) were seen to pursue abnormal cytokinesis with triple or quadruple midbodies (Figure 3B, i). These aberrant cell divisions result into several (more than two) cells that are usually aneuploid (Figure 3B, j, k). Asymmetric distribution of cytoplasm and chromatin material was observable showing separation into more than two abnormal cells (even multinucleate) suggesting high degree of aneuploidy caused by drug treatment (Figure 3B, l, m). Asymmetric cytokinesis following multipolar mitosis generated three or more aneuploid daughter cells that may perhaps be inviable. In addition, several fragmented DNA pieces and clusters of small apoptotic bodies were seen at 48 hrs post-treatment (Figure 3B, n).

### EM011 treatment induced mitotic exit after the transient mitotic block

**Figure 4:**
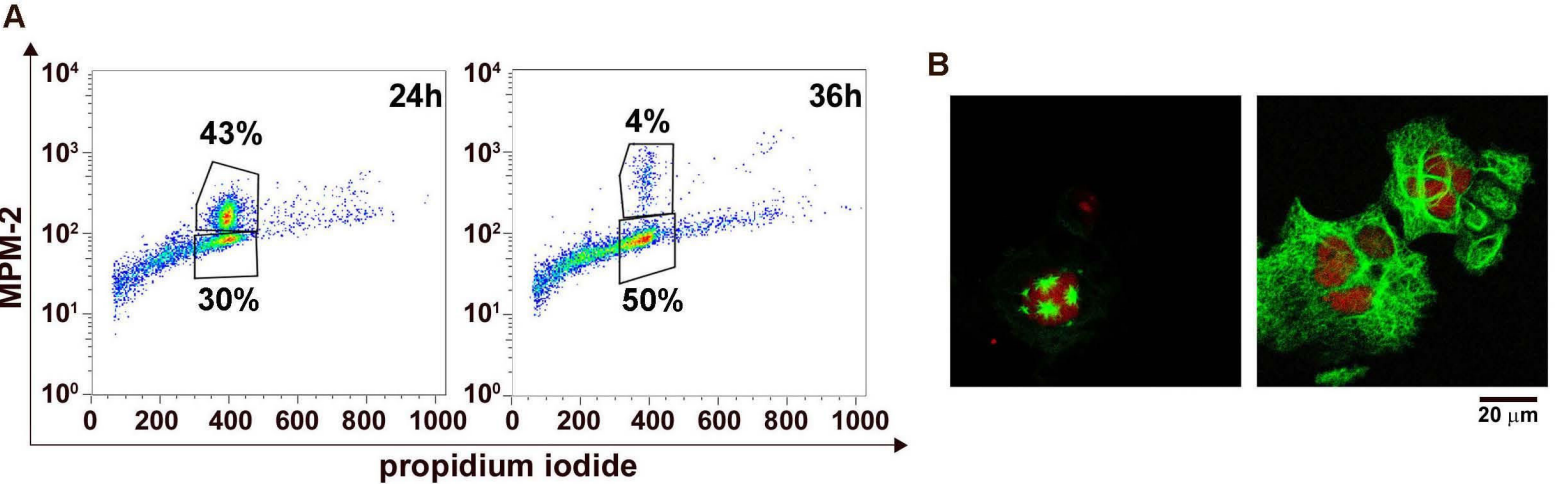
**A. Quantitative flow-cytometric representation of mitotic exit using two-color staining (MPM-2 and PI)**. Representative dot-plot shows that the mitotic population plummets at 36 hrs of EM011 treatment and there is a substantial increase in the 4N G2 population suggesting mitotic exit. **B**. Representative confocal immunofluorescence images of microtubules (green) and DNA (red) showing a multipolar mitotic cell (left panel) and a mitotically-exited G1-like multinucleate cell (right panel).

We next quantitated the cell population that slipped out of mitosis without cytokinesis using dual color-flow cytometry (MPM-2 and PI). There was a significant drop in the percentage of MPM-2 positive cells from ~43% (44 ± 3%) at 24 hrs to ~4% (4.2 ± 0.5%) at 36 hrs of EM011 treatment indicating that cells had slipped out of mitosis (Figure 4A). After mitotic exit, this population of cells then appears as the MPM-2 negative tetraploid (4N) population that increased from ~30% (30.4 ± 2.2%) at 24 hrs to ~50% (49.6 ± 3.2%) at 36 hrs (Figure 4A). These flow-cytometric data correlated with confocal microscopic observations, lending support to the fact that cells with aberrant multipolar spindles stall transiently in mitosis, followed by decondensation of chromatin material, and an exit from mitotic phase. Since cells have failed to successfully progress through mitosis to execute cytokinesis, they have 4N DNA amounts and can be seen as huge multinucleated cells. Figure 4B depicts representative cells from both states, mitotically-arrested (left panel) and pseudo G1-like multinucleate state (right panel).

### EM011 treatment alters expression of cell-cycle and apoptosis regulatory molecules

#### EM011 activates spindle assembly checkpoint in A549 cells

**Figure 5:**
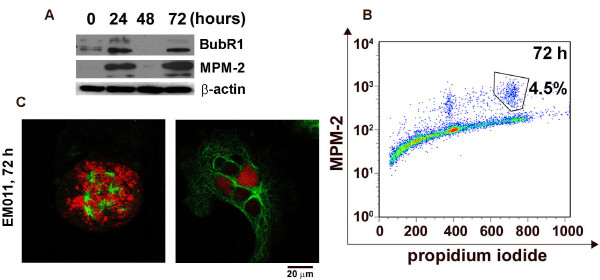
**EM011 induces mitotic arrest by the activation of the spindle checkpoint**. **A **shows immunoblots for BubR1 and MPM-2. Uniform loading of lysates was confirmed by immunoblotting for actin. **B**. Cells were analyzed for mitotic population at 72 hrs post-treatment using two-color flow cytometry. Representative dot-plot shows a clear 8N mitotic population (MPM-2 positive population) suggesting that mitotically exited cells continue to progress in the cell-cycle and enter a second round of DNA replication and mitosis. **C **shows an abnormally massive mitotic cell at 72 hrs (left) and a significantly huge G1-like multinucleate cell (right).

Most antimitotics are known to activate the spindle assembly checkpoint [23]. BubR1, a component of the mitotic checkpoint is phosphorylated during spindle checkpoint activation [26]. Immunoblotting data showed that EM011 activated the mitotic checkpoint as detected by phosphorylation of BubR1 at 24 hrs of drug treatment (Figure 5A). Intriguingly, the BubR1 signal faded at 48 hrs and reappeared at 72 hrs post-treatment (Figure 5A). Probing with MPM-2 antibody, a mitosis-specific marker, that recognizes phosphorylation of Ser/Thr-Pro epitopes, also showed re-emergence of positive MPM-2 signals at 72 hrs post-treatment (Figure 5A). The BubR1 phosphorylation at 24 hrs indicated the initial activation of the spindle assembly checkpoint that accompanied drug-induced mitotic arrest. Since mitotic slippage occurs after prolonged mitotic arrest (beyond 24 hrs), it is reasonable to speculate that the absence of MPM-2 signals indicates exit from mitosis at 48 hrs. However, the absence of BubR1 signals at 48 hrs is intriguing because mitotic slippage from aberrant mitosis usually takes place by overriding an activated checkpoint [23,26]. Both microscopic and flow-cytometric data show that A549 cells exited mitosis after a prolonged mitotic arrest upon activation of the spindle checkpoint. To investigate the re-emergence of BubR1 and MPM-2 signals, A549 cells were drug-treated for 72 hrs, stained using PI and MPM-2 followed by dual-color flow-cytometric analyses (Figure 5B). An emergence of a MPM-2 positive cell population (4.9 ± 0.6%) with 8N DNA amounts suggested that a sub-population of 'mitotically slipped-out' cells perhaps, continue the cell-cycle by entering another round of DNA replication and mitosis. Parallel immunostaining experiments revealed that 72 hrs drug-treated cells showed some highly multipolar chaotic mitotic figures (Figure 5C, left) as well as excessively huge G1-like multinucleated/multilobed cells (Figure 5C, right). It is noteworthy that BubR1 and MPM-2 signals reappear at 72 hrs and at this point of time, the cells are much larger in size compared with the first mitosis which is consistent with cell growth without cell division. We speculate that the massive cells with excessively huge DNA contents perhaps, eventually trigger apoptosis due to genotoxic stress.

#### EM011 downregulates survivin expression in A549 cells

**Figure 6:**
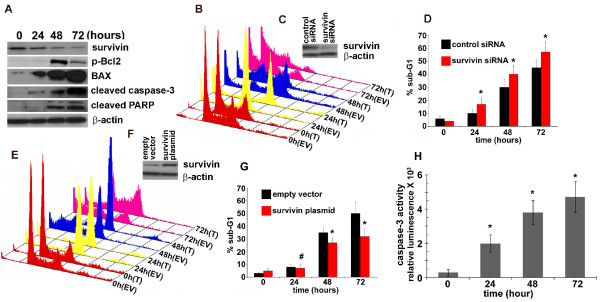
**A. Immunoblot analysis of A549 cells treated with EM011 for 0, 24, 48 and 72 hrs**. After the indicated times, cells were lysed and total protein was extracted, separated by SDS-PAGE, electrotransferred onto polyvinylidene difluoride membrane, and subjected to immunoblotting with the indicated primary antibodies followed by incubation with horseradish peroxidase-conjugated secondary antibodies. β-actin was used as a loading control. **B**. Knock-down of survivin using survivin siRNA enhances the apoptotic response of A549 cells to EM011 treatment. A549 cells were transfected with a plasmid encoding survivin siRNA and were then subjected to EM011 exposure for 0, 24, 48 and 72 hrs. An empty vector control was used in the experiments. Panel **B **shows three-dimensional FACS profile of survivin siRNA-transfected (T) and empty vector transfected (EV) cells for 0, 24, 48 and 72 hrs. **C**. Western blot analysis of the expression of survivin and β-actin in A549 cells transfected with control or survivin siRNAs. **D**. Quantitation of the percent sub-G1 population for control siRNA and survivin siRNA transfected cells. Values represent averages and error bars show SD (*, p < 0.05). **E**. Overexpression of survivin renders A549 cells resistant to EM011-induced apoptosis. A549 cells were transfected with a survivin encoding plasmid construct to ectopically overexpress survivin. Panel **E **shows cell-cycle profiles of survivin-transfected cells along with empty vector-transfected controls. **F**. Western blot analysis of the expression of survivin and β-actin in A549 cells transfected with control or survivin plasmid. **G**. Quantitation of the percent sub-G1 population for the survivin-transfected and empty vector-transfected controls. Values represent averages and error bars show SD (^#^, not significant; *, p < 0.05). **H**. Quantitation of the time-dependent increase in caspase-3 activity upon EM011 treatment. Cells were treated with 25 μM EM011 for 0, 24, 48, and 72 hrs, and caspase-3 activity was analyzed using the luminogenic substrate Z-DEVD-aminoluciferin. The values and error bars shown in the graph represent the averages and standard deviations, respectively, of three independent experiments; (p < 0.05).

Survivin, an antiapoptotic member of the inhibitor of apoptosis (IAP) family is known to block apoptosis by inhibiting caspases and antagonizing mitochondria-dependent apoptosis [27]. A decrease in survivin levels plays an important role in human NSCLC and in agreement with this notion; several studies have concluded that there is a strong correlation between increased survivin levels and progression of human lung cancer [28-30]. Survivin is expressed at high level in many types of cancers, but not in normal tissues from the same organs [31]. We thus asked if EM011 alters survivin levels as part of its anti-proliferative and pro-apoptotic action. To this end, the expression levels of survivin were examined upon EM011 treatment over time. 25 μM EM011 caused a decline in survivin levels as early as 24 hrs and significantly low levels were seen at 72 hrs post treatment (Figure 6A). In contrast, H1792 cells with a high IC_50 _value (~50 μM) for EM011, did not show change in survivin levels over time of drug treatment (see Additional File 1), suggesting that survivin may play a role in conferring resistance to EM011-induced apoptosis.

#### Knock-down of endogenous survivin by survivin siRNA sensitizes A549 cells to EM011-induced apoptosis

If survivin is important to EM011-induced apoptosis, knock-down of endogenous survivin expression should render A549 cells to become more susceptible to EM011 treatment. Thus, to determine whether downregulation of survivin contributes to EM011-induced apoptosis, we used a plasmid construct encoding survivin specific siRNA to selectively knock-down endogenous survivin in A549 cells. After the knock-down, cells were drug-treated for 24, 48 and 72 hrs. Downregulation of survivin sensitized A549 cells to EM011-induced apoptosis, whereas control siRNA had no effect. Figure 6B shows representative cell-cycle profiles indicating that abrogation of survivin led to an increased sensitivity of lung cancer cells towards apoptosis upon drug treatment. This is evident from the differences in percent sub-G1 populations in cells that were EM011-treated for 48 or 72 hrs after survivin knock-down (~40% at 48 hrs and ~57% at 72 hrs) versus cells subjected to control siRNA transfections (~30% at 48 hrs and ~45% at 72 hrs) prior to EM011 exposure for the matched time points (Figure 6D). Immunoblots showing knock-down efficiency of survivin siRNA at 24 hrs post-transfection compared to survivin levels in cells treated with control siRNA for the same time are depicted in Figure 6C.

#### Overexpression of survivin renders A549 cells resistant to EM011-induced apoptosis

To further confirm the role of survivin in EM011-induced apoptosis, we overexpressed survivin in A549 cells using a survivin encoding plasmid construct. Ectopic overexpression of survivin protected cells against EM011-induced apoptosis, whereas, control plasmid (empty vector, EV) had no effects. Figure 6E depicts cell-cycle profiles showing in pairs, the control plasmid (EV) and survivin plasmid transfected (T) cells, which were drug-treated for 24, 48 and 72 hrs post-transfection. There was a reduction in percent sub-G1 population in cells that were transfected with the survivin plasmid (~27% at 48 hrs) compared to when cells were transfected with a control plasmid construct (~35% at 48 hrs). The protective effects of survivin overexpression were also visible at 72 hrs of transfection (~32% sub-G1 population) compared to control plasmid (~50% sub-G1 population) (Figure 6G). However, among the control and survivin plasmid transfected cells, differences in sub-G1 population at 24 hrs post drug-treatment were not statistically significant. Immunoblots showing ectopic overexpression efficiency of the survivin plasmid at 24 hrs post-transfection compared to survivin levels in cells treated with empty pcDNA3 vector are depicted in Figure 6F.

#### EM011 decreases Bcl2/BAX ratio in A549 cells

It has been proposed that prolonged mitotic arrest stimulates the phosphorylation of Bcl2, thereby resulting in its inactivation [32-34]. Bcl2 in the unphosphorylated form complexes with BAX, and thus its phosphorylation releases BAX from the Bcl2-BAX complex [35-37]. Unbound BAX translocates from cytosol to the mitochondrial membrane to signal triggering of the downstream apoptotic cascade, such as release of cytochrome c and activation of executionery caspases [35-37]. EM011 activates mitochondrially-mediated intrinsic apoptotic pathway in breast cancer and lymphoma cells [14,15,17]. To examine the time-dependent effects of EM011 on Bcl2 proteins in A549 cells, we analyzed changes in the expression levels of proapoptotic BAX and antiapoptotic Bcl2. EM011 induced mitotic arrest in A549 cells was accompanied by the hyperphosphorylation of Bcl2 and there occurred an increase of BAX protein levels in a time-dependent manner (Figure 6A). This led to a decrease in the antiapoptotic/proapoptotic (Bcl2/BAX) ratio as a function of time of treatment (Figure 6A) suggesting involvement of Bcl2 family members in EM011-induced cell death.

#### EM011 causes activation of caspase-3 and cleavage of its downstream target, PARP

Upon cleavage by upstream proteases in an intracellular cascade, the activation of caspase-3 is considered as a hallmark of the apoptotic process. The levels of cleaved active subunits of executioner caspase-3 were evaluated by immunoblotting cell lysates following EM011 treatment for 0, 24, 48 and 72 hrs. EM011 caused a significant increase in activated caspase-3 following 72 hrs of EM011 exposure (Figure 6A). To confirm the involvement of caspase-3, the active form of the cysteine protease was monitored using a small conserved modified peptide substrate that becomes luminogenic upon cleavage. EM011 treatment caused a time-dependent activation of caspase-3 in A549 cells (Figure 6H). Next, we examined the activation-mediated cleavage of caspase-3 substrate, poly(ADP- ribose) polymerase (PARP), which is a reliable marker of apoptosis. Utilizing their cysteine protease activity, caspases separate N-terminal DNA-binding domain of PARP from its C-terminal catalytic domain (89 kDa) [38]. A time-dependent increase in cleaved PARP was observed upon probing with a cleaved PARP specific antibody (Figure 6A). Overall, these results show activation of caspase-3 and PARP cleavage suggesting that EM011 induced apoptotic cell death in A549 cells.

#### EM011 triggers apoptosis as seen by an increase of TUNEL-positive cells

**Figure 7:**
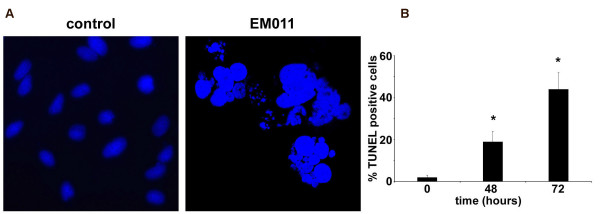
**EM011 treatment induces DNA fragmentation and apoptosis**. **A **shows DAPI-stained micrographs for control (left) and 72 hrs drug exposed (right) A549 cells using a 63× objective. **B**. Quantitation of percent TUNEL-positive cells for EM011-treated cells for 0, 48 and 72 hrs. Values, mean of three independent experiments; bars, SD; (*, p < 0.05).

Figure 7A shows DAPI-stained micrographs with fragmented nuclei reminiscent of apoptotic bodies at 72 hrs post-treatment, emphasizing the incidence of apoptosis. The percentage of cells with condensed and fragmented nuclei increased with the time of drug treatment (data not shown). To further validate apoptosis, the increase in the concentration of 3'-DNA ends due to fragmentation was quantified using a flow-cytometry based TUNEL assay. EM011-treated A549 cells showed ~44% TUNEL-positive cells (Figure 7B) at 72 hrs of exposure compared to control cells, suggesting extensive DNA cleavage.

## Conclusion

These data show that EM011 is anti-proliferative and pro-apoptotic in lung cancer cells. The inhibition of cellular proliferation is perhaps due to induction of a robust transient mitotic arrest in A549 cells. This is followed by an abnormal exit of cells from mitosis without cytokinesis into a pseudo G1-like multinucleate state. These abnormally huge cells perhaps trigger activation of apoptotic cell death program that is mitochondrially-driven and executed through the activated caspase machinery by the cleavage of downstream targets such as PARP. The A549 cell death program is also mediated through downregulation of survivin, in that knock-down of survivin sensitized cells to undergo apoptosis whereas overexpression of survivin reduced EM011-induced apoptosis. This study identifies the potential usefulness of EM011, a non-toxic microtubule-modulating agent, in the management of lung cancer.

## Competing interests

The authors declare that they have no competing interests.

## Authors' contributions

PK carried out most experiments, and contributed towards drafting the manuscript. SS contributed towards performing cell proliferation studies. CY participated in analysis of microscopy data and helped in statistical analysis. SP participated in design of study. RA conceived, designed and coordinated the study, performed experiments and drafted the manuscript. All authors read and approved the final manuscript.

## Supplementary Material

Additional file 1 **Immunoblot analysis of survivin expression in H1792 lung cancer cells**. Representative immunoblot analysis of survivin expression in H1792 lung cancer cells treated with EM011 for 24, 48 and 72 hrs. Actin was used as a loading control. Click here for file
