# Supplementary material for: EM011 activates a survivin-dependent apoptotic program in human non-small cell lung cancer cells
Source: Mol Cancer. 2009 Oct 30;8:93. doi: 10.1186/1476-4598-8-93 (PMC2776016; doi:10.1186/1476-4598-8-93)
Supplement: Additional file 1 — Immunoblot analysis of survivin expression in H1792 lung cancer cells. Representative immunoblot analysis of survivin expression in H1792 lung cancer cells treated with EM011 for 24, 48 and 72 hrs. Actin was used as a loading control. [file 1476-4598-8-93-S1.docx]

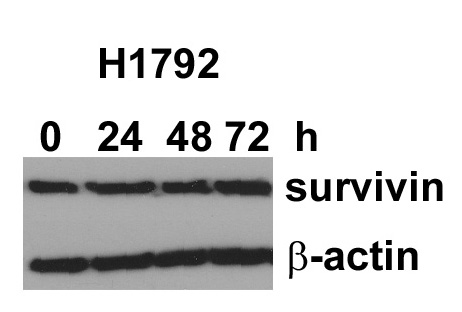


Representative immunoblot analysis of survivin expression in H1792 lung cancer cells treated with EM011 for 24, 48 and 72 hrs. Actin was used as a loading control.
